# Air-tolerant solar reforming of pre-treated biomass and plastics in viscous sustainable solvents

**DOI:** 10.1039/d6sc01540a

**Published:** 2026-05-11

**Authors:** Dongseok Kim, Papa K. Kwarteng, Erwin Reisner

**Affiliations:** a Yusuf Hamied Department of Chemistry, University of Cambridge Lensfield Road Cambridge CB2 1EW UK reisner@ch.cam.ac.uk

## Abstract

We report an air-tolerant solar reforming (SR) system for the valorisation of pre-treated biomass and plastic waste streams. This process operates in low O_2_-diffusivity solvents such as the robust deep eutectic solvent (DES) ZnCl_2_-acetamide, which prevents O_2_ from interfering with photocatalysis by avoiding easily oxidisable components (*e.g.* alcohols) in water. This approach sustains SR performance under aerobic conditions using a carbon nitride photocatalyst, enabling photooxidative valorisation of depolymerised cellulose and polyethylene terephthalate (PET) while generating H_2_ fuel. SR using low O_2_-diffusivity solvents is scalable and also adaptable to non-innocent viscous liquid wastes such as glycerol, which can act as both the low O_2_-diffusivity solvent to exclude O_2_ and the organic waste source to produce valuable chemicals.

## Abstract 2

We demonstrate an air-tolerant photocatalytic system for solar reforming using a low oxygen-diffusivity solvent/water mixture. This approach sustains photocatalytic performance under air, enabling biomass and plastic waste valorisation while generating hydrogen fuel.

## Graphical abstract

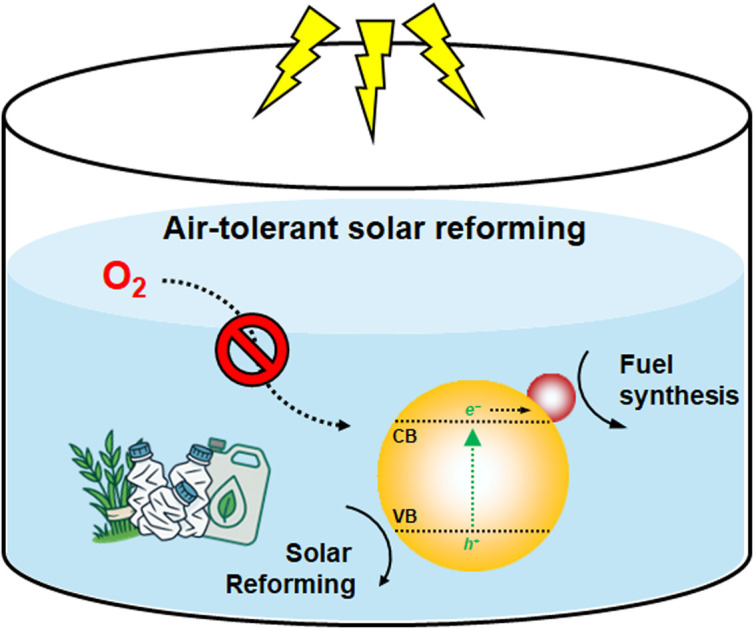

## Introduction

Photocatalytic H_2_ production using solar energy is a promising strategy to produce sustainable fuel as an alternative to conventional carbon-intensive H_2_ production methods.^1,2^ While overall water splitting offers a pathway for clean H_2_ production, its practical implementation has been hampered by its highly endothermic reaction and co-evolution of O_2_ with limited commercial value.^3^ Solar reforming (SR) employs waste streams such as biomass or synthetic polymers (plastics) as electron donors, thereby lowering the energy barrier of the reaction, enhancing value creation by co-producing organic chemicals from waste oxidation without O_2_ evolution and thus improving commercial prospects.^4,5^

The production of H_2_ through SR has therefore received significant attention, but the process requires anaerobic conditions as O_2_ (air) is known to have a detrimental effect on photocatalytic performance. (Photo)catalytic H_2_-evolution systems perform generally poorly in the presence of O_2_ due to the thermodynamically favourable O_2_ reduction reaction to produce reactive oxygen species (ROS) unless specific protection mechanisms are in place.^6^ Thus, inert atmospheres such as N_2_ or Ar are commonly employed in photo(electro)catalytic H_2_ production. However, tolerance to air is practically important as some exposure will be unavoidable in commercial or industrial deployment.^7^ While the development of O_2_-tolerant solid-state,^8^ metal complex,^9,10^ and enzymatic H_2_ catalysts^11,12^ is an active area of research, it remains unexplored in SR.

Deep eutectic solvents (DESs) have emerged as a novel class of green solvents characterised by their ease of preparation, low cost, and recyclability as well as their tuneable physicochemical properties (*e.g.*, negligible vapour pressure), and environmental friendliness (*e.g.*, biodegradability).^13,14^ DESs are typically formed through the complexation of a hydrogen bond donor and a hydrogen bond acceptor, often involving readily available components such as choline chloride and urea. DESs exhibit melting points significantly lower than those of their individual constituents due to strong hydrogen bonding interactions. Their unique solvent properties have enabled widespread applications across diverse fields, including catalysis,^15,16^ electrochemistry,^17,18^ and materials synthesis,^19^ thereby positioning DESs as versatile media for green and sustainable chemistry.

It has recently been demonstrated that O_2_-sensitive methyl viologen radicals can be stably generated *via* electrochemical, chemical, and photochemical methods and retained in DESs under air.^20^ This ultrastability is attributed to the significantly reduced O_2_ diffusivity in DESs, which protects the radicals from oxidative degradation under aerobic conditions. Low O_2_-diffusivity also led to the development of air-tolerant redox flow batteries by using pyridinium-based electrolytes, effectively mitigating capacity fade caused by reduced radical species.^21^ These findings highlight the possibilities of developing air-tolerant solvent systems with potential impact across various applications. Various DESs have also been used to demonstrate O_2_-tolerant H_2_ production,^22,23^ and CO_2_ reduction,^24^ but these photocatalytic systems required easily-oxidisable sacrificial electron donors (*e.g.*, triethanolamine) as the DESs contained primary alcohols which can be readily oxidised and thereby decomposed under photocatalytic conditions. To extend this strategy for practical depolyment in SR processes, robust SR systems with DESs resistant to oxidative decomposition during the photocatalytic cycle are required.

**Scheme 1:**
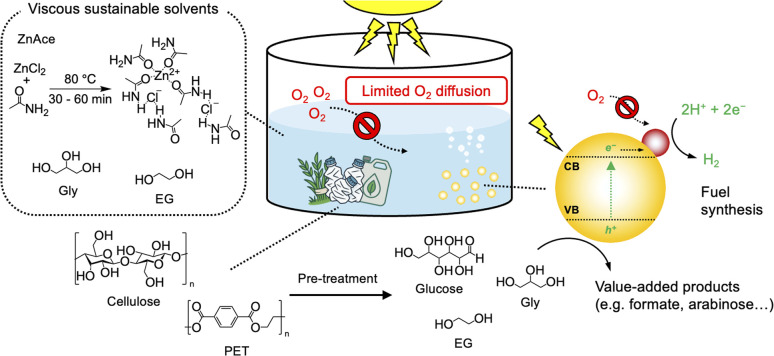
Schematic illustration of air-tolerant SR of pre-treated cellulose and PET with a platinised carbon nitride photocatalyst using viscous sustainable solvents such as ZnAce, glycerol (Gly), or ethylene glycol (EG).

Here, we report a DES, composed of ZnCl_2_ and acetamide (denoted as ZnAce), which is suitable for aerobic SR of polymeric waste streams. ZnAce, which avoids the presence of easily oxidisable primary alcohols commonly present in widely used DESs, exhibits high stability during SR cycles and thereby enables the selective, challenging oxidation of waste substrates without undergoing oxidation itself. SR in aqueous ZnAce thereby facilitates air-tolerant SR of pre-treated biomass and plastics to produce H_2_, maintaining SR performance under air without a significant loss in activity (Scheme 1). We further show that aerobic SR can be readily scaled up. Air-tolerant SR can even be achieved under practical conditions using viscous liquid waste steams such as glycerol and ethylene glycol, which act directly as the low O_2_-diffusivity solvent and organic substrate without DES. Our approach thus offers a versatile and scalable platform for aerobic operation of SR in the future.

## Results and discussions

Cyanamide-functionalised carbon nitride modified with a platinum H_2_ evolution co-catalyst was prepared and characterised as previously reported,^25,26^ and used as a photocatalyst in this study (see experimental Section, Fig. S1). The platinum co-catalyst also displays a well-established activity in catalysing the O_2_ reduction reaction, which helps to investigate the performance of ZnAce in promoting O_2_-tolerance of the dispersed photocatalyst solution when exposed to aerobic conditions. ZnAce was prepared following a previously reported procedure by stirring a mixture of ZnCl_2_ and acetamide (1 : 4 molar ratio) at 80 °C until a homogeneous liquid was formed.^27^

SR was optimised in the robust DES, ZnAce, which is free of readily oxidisable primary alcohols typical of many DESs, and carried out in a glass reactor with full solar spectrum irradiation (100 mW cm^−2^, AM 1.5G) at 25 °C in a 3 mL solution of ZnAce with an aqueous solution containing glucose (50 mg mL^−1^, ∼280 mM) and phosphate buffer (KP_i_, 10 mM, pH 4.5). Mildly acidic conditions were selected because the aqueous ZnAce solution precipitated under alkaline conditions (pH ≥ 7), most likely due to disruption of the ionic interactions between ZnCl_2_ and acetamide (Fig. S2). Glucose is the soluble monomeric building block of cellulose and was therefore selected as a model electron donor. The glass reactor was charged with a solution containing the platinised carbon nitride (5 mg, Pt-loading 0.60 ± 0.03 wt%), sealed with a rubber septum to collect the produced H_2_, and the headspace contained air for aerobic SR or N_2_ containing 2% CH_4_ as an internal standard for anaerobic SR. The produced H_2_ was periodically monitored by analysing the headspace (4.8 mL) of the photoreactor using gas chromatography.

**Fig. 1:**
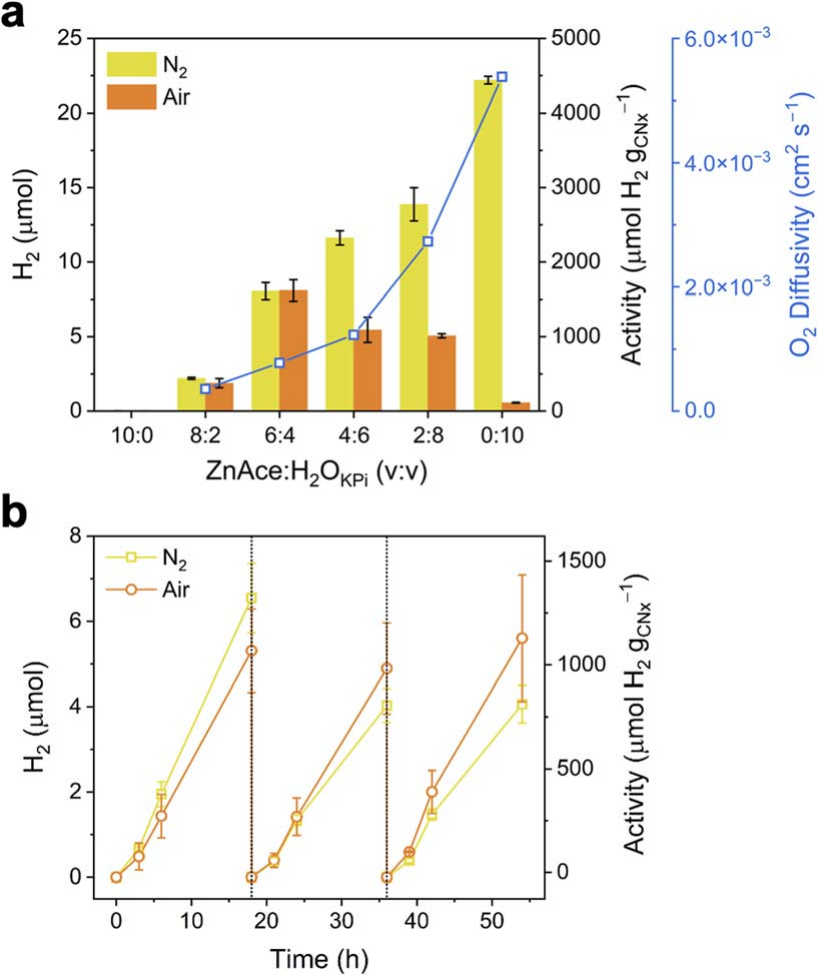
SR performance under N_2_ or air using a platinised carbon nitride photocatalyst with glucose (50 mg mL^−1^) as an electron donor in ZnAce : H_2_O_KP_i__ (H_2_O contains 10 mM KP_i_ at pH 4.5) under simulated solar light irradiation (100 mW cm^−2^, AM 1.5G) at 25 °C. (a) H_2_ generation after 18 h and O_2_-diffusivity as a function of the ZnAce : H_2_O_KP_i__ ratio. (b) Glucose SR performance with photocatalyst recycling every 18 h, where the supernatant was removed and fresh ZnAce:H_2_O_KP_i__ (6 : 4 v/v) solution and substrate added.

To investigate the effect of ZnAce on H_2_ production, the SR performance was initially screened under N_2_ by varying the reaction medium composition between ZnAce and the KP_i_ buffered aqueous solution (Fig. 1a and S3). The activities under N_2_ gradually increased with the addition of water due to the improved supply of protons. However, a different trend was observed under air, where adverse effects on photocatalysis emerged above 60% of H_2_O_KP_i__ contents with negligible H_2_ production in pure aqueous solution (see below for details). Importantly, the SR performance under air was nearly comparable with the N_2_ purged system up to 40% of H_2_O_KP_i__ content, with a maximal activity of 1610 ± 114 µmol H_2_ per g_CN_*x*__ (53.7 ± 3.80 µmol H_2_ per g_substrate_) under N_2_ and 1620 ± 143 µmol H_2_ per g_CN_*x*__ (54.0 ± 4.78 µmol H_2_ per g_substrate_) under air after 18 h of SR in ZnAce : H_2_O_KP_i__ (6 : 4 v/v) solution (see Table S1 for literature comparison). The marginal differences in SR performance under N_2_ and air implied that atmospheric O_2_ did not significantly hamper the catalytic cycle of the photocatalyst in ZnAce.

Exclusion control experiments in air and N_2_ showed no or negligible amounts of H_2_ in the absence of electron donor, co-catalyst or light, verifying that ZnAce was not involved in the SR process (Table 1).

A comparison experiment was executed to identify the oxidative stability of different types of DESs under the oxidatively demanding conditions of SR using the photocatalyst. The most widely used DESs contain easily oxidisable alcohols (reline: choline chloride and urea; ethaline: choline chloride and ethylene glycol; glyceline: choline chloride and glycerol; Fig. S4), and a significant amount of H_2_ was detected during irradiation even in the absence of an external electron donor, indicating that these DESs oxidatively decompose and are not suitable for SR (Fig. S5). This is supported by linear sweep voltammograms (LSV) of DES : H_2_O_KP_i__ (6 : 4 v/v) solutions under N_2_, which demonstrate that ZnAce is more stable under oxidative conditions (anodic potential) compared to other DESs (Fig. S6). These results highlight that ZnAce provides a uniquely robust medium for air-tolerant SR of waste streams in the aqueous DES.

**Table 1:** Exclusion control experiments for SR of glucose (50 mg mL^−1^) in ZnAce : H_2_O_KP_i__ (6 : 4 v/v; H_2_O contains 10 mM KP_i_ at pH 4.5) under N_2_ or air using a platinised carbon nitride photocatalyst under simulated solar light irradiation (100 mW cm^−2^, AM 1.5G) at 25 °C after 18 h. Blank (−) means the number of produced gases was less than the limit of detection (0.01 µmol)

	N_2_			Air		
	H_2_ [µmol]	Activity [µmol H_2_ per g_CN_*x*__]	Yield [µmol H_2_ per g_Substrate_]	H_2_ [µmol]	Activity [µmol H_2_ per g_CN_*x*__]	Yield [µmol H_2_ per g_Substrate_]
Full system	8.05 ± 0.57	1610 ± 114	53.7 ± 3.80	8.09 ± 0.72	1620 ± 143	54.0 ± 4.8
No SED	0.04 ± 0.00	8.89 ± 0.06	0.30 ± 0.01	0.031 ± 0.01	6.18 ± 2.00	0.21 ± 0.07
No Pt	0.05 ± 0.01	10.3 ± 1.25	0.34 ± 0.04	0.04 ± 0.01	7.89 ± 0.91	0.26 ± 0.03
No light	—	—	—	—	—	—

Molybdenum disulfide (MoS_2_) was also employed as co-catalyst instead of the precious-metal Pt for SR of glucose, and exhibited similar behaviour to Pt without significant decrease in performance under air compared to N_2_ (Fig. S7).

To elucidate the origin of the air-tolerant SR, several physicochemical properties of the ZnAce : H_2_O_KP_i__ solutions, O_2_ solubility, viscosity, and O_2_ diffusivity, were systematically investigated. As summarised in Table 2 and Fig. S8, increasing the ZnAce content led to a noticeable rise in viscosity, accompanied by an increase in O_2_ solubility. Notably, the O_2_ diffusivity calculated from the Levich equation (see experimental Section in SI for details) decreased as the ZnAce concentration increased, suggesting that suppressed O_2_ mass transport is a key factor enabling air-tolerant SR (Fig. 1a). This observation is consistent with the Stokes–Einstein relationship, where higher solution viscosity results in reduced molecular diffusion. Accordingly, the increased viscosity of the ZnAce : H_2_O_KP_i__ solution restricts O_2_ diffusion, thereby limiting the oxygen reduction reaction (ORR) kinetics and effectively facilitating SR under aerobic conditions. At the same time, however, viscosity is also closely associated with proton diffusion; consequently, the H_2_ production activity decreases as viscosity increases. These opposing effects reveal an inherent trade-off between minimising ORR and maintaining efficient H_2_ production. On the basis of this balance, the optimal reaction condition was identified as a 6 : 4 volume ratio of ZnAce : H_2_O_KP_i__.

**Table 2:** Kinematic viscosities and O_2_ diffusivities at 1 atm air and O_2_ solubilities under O_2_ saturated conditions in the ZnAce : H_2_O_KP_i__ solutions

ZnAce : H_2_O_KP_i__ (v/v)	Kinematic viscosity (cm^2^ s^−1^)	O_2_ solubility (mmol L^−1^)	O_2_ diffusivity (cm^2^ s^−1^)
0 : 10	1.07 ± 0.032 × 10^−2^	0.121 ± 0.047	5.38 × 10^−3^
2 : 8	2.88 ± 0.040 × 10^−2^	0.222 ± 0.036	2.73 × 10^−3^
4 : 6	4.84 ± 0.104 × 10^−2^	0.361 ± 0.040	1.23 × 10^−3^
6 : 4	6.59 ± 0.040 × 10^−2^	0.469 ± 0.040	7.78 × 10^−4^
8 : 2	8.53 ± 0.040 × 10^−2^	0.653 ± 0.045	3.58 × 10^−4^

To directly validate the limited ORR during SR, O_2_ levels were monitored in the headspace to confirm O_2_ consumption during glucose SR (Fig. S9). O_2_ reduction is competing with proton reduction and occurs substantially in pure H_2_O, causing 48% of O_2_ consumption (∼21.5 µmol, which is comparable to the amount of generated H_2_ from SR under N_2_) after 24 h. In contrast, only 7.0% of O_2_ was consumed in the headspace above the ZnAce : H_2_O_KP_i__ (6 : 4 v/v) solution. These data support that O_2_ cannot effectively diffuse in the aqueous ZnAce medium, and the solvent thereby protects the photocatalyst from O_2_ exposure, thereby enabling efficient photocatalytic H_2_ production under aerobic conditions.

The oxidation products were determined by high-performance liquid chromatograph (HPLC) and ^1^H nuclear magnetic resonance (NMR) spectroscopy after SR of glucose with the platinised carbon nitride photocatalyst in ZnAce : H_2_O (6 : 4 v/v) mixture (Fig. S10). The main products of glucose SR in ZnAce/H_2_O_KP_i__ were arabinose (C_5_H_10_O_5_) and formate (HCOO^−^), which is consistent with the results of SR in H_2_O under N_2_.^28^ Although the quantified oxidation products were not stoichiometrically matched with the reduced products (*ca.* 2 : 1 Red : Ox), we speculate that formate may undergo further oxidation to CO_2_,^29^ which could account for the observed stoichiometry. Importantly, the formation of arabinose and formate indicates that ZnAce does not alter the reaction selectivity or directly participates in the photocatalytic SR cycle, but instead primarily regulates O_2_ diffusion, thereby enabling air-tolerant SR.

The platinised carbon nitride photocatalyst could be retrieved through centrifugation and reused by re-suspending in a fresh ZnAce : H_2_O_KP_i__ solution, followed by glucose SR under simulated solar light. The recycled photocatalyst maintained over 60% of its activity under N_2_ and nearly 100% under air of its SR activity compared to the previous 18 h irradiation interval (Fig. 1b). To further assess the robustness of the photocatalyst in the aqueous ZnAce medium, a 5 day continuous stability test was performed under the simulated solar light irradiation (Fig. S11). No noticeable decline in the H_2_ production rate was observed throughout the extended SR operation, demonstrating the sustained stability of the air-tolerant SR system.

X-ray Photoelectron Spectroscopy (XPS) and Inductively Coupled Plasma Optical Emission Spectroscopy (ICP-OES) of the photocatalyst, following washing and drying after 18 h of SR, revealed some photo-deposition of Zn^0^ and surface adsorption of Zn^2+^ ions on the carbon nitride (Fig. S12). In addition, transmission electron microscopy coupled with energy-dispersive X-ray (TEM-EDX) analysis also confirmed the presence of Zn on the catalyst surface, but no significant structural damage or detrimental changes to the photocatalyst were observed (Fig. S13). Despite the unexpected Zn-deposit on the photocatalyst, the retained recyclability under air, structural integrity, and long-term stability of the photocatalyst indicated that ZnAce did not exert a detrimental influence on SR performance and catalyst recyclability.

**Fig. 2:**
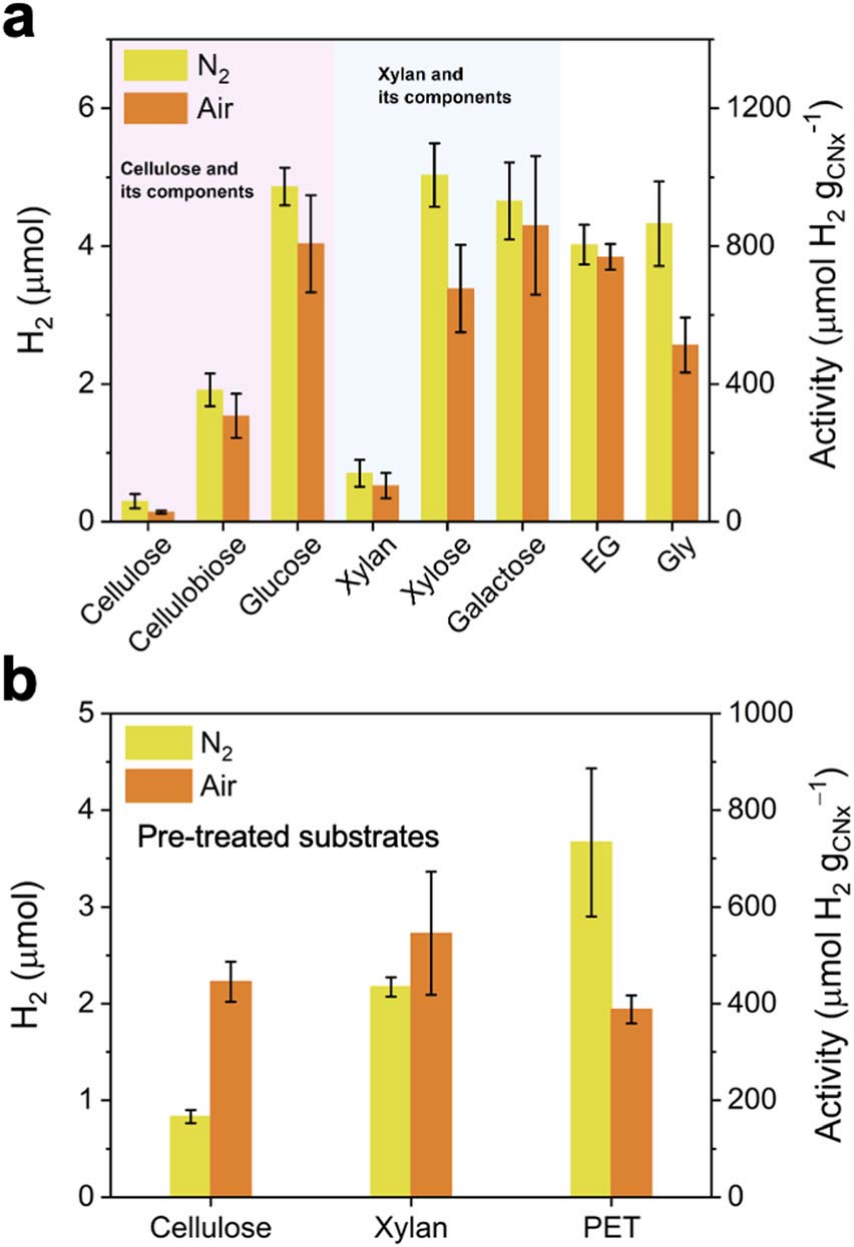
(a) SR performance coupled with H_2_ production after 18 h under N_2_ or air using platinised carbon nitride photocatalyst in 3 mL of ZnAce : H_2_O_KP_i__ (10 mM, pH 4.5) in H_2_O (6 : 4 v/v) for lignocellulosic components, EG, and Gly (100 mg). (b) SR performance after 18 h under N_2_ or air using pre-treated cellulose, xylan and PET in 3 mL of ZnAce: 0.75 M H_2_SO_4_ (6 : 4 v/v) solution under N_2_ and air. All experiments were conducted under simulated solar light irradiation (100 mW cm^−2^, AM 1.5G) at 25 °C.

Air-tolerant SR (100 mg of substrates) into H_2_ using a ZnAce : H_2_O_KP_i__ (6 : 4 v/v) mixture was subsequently studied using polymeric substrates, *viz*. cellulose and xylan, along with their soluble substrates (Fig. 2a).^30^ As the solubility of the substrates in ZnAce/H_2_O_KP_i__ increases, improved photocatalyst-substrate contact leads to significantly enhanced SR activity. After 18 h of simulated solar light irradiation, xylose produced 1010 ± 92 µmol H_2_ per g_CN_*x*__ under N_2_ and 677 ± 127 µmol H_2_ per g_CN_*x*__ under air, and galactose generated 932 ± 112 µmol H_2_ per g_CN_*x*__ under N_2_ and 860 ± 202 µmol H_2_ per g_CN_*x*__ under air. The SR performance for insoluble, polymeric α-cellulose and xylan was 59.6 ± 20.7 µmol H_2_ per g_CN_*x*__ under N_2_ and 27.6 ± 4.43 µmol H_2_ per g_CN_*x*__ under air, and 141 ± 39.2 µmol H_2_ per g_CN_*x*__ under N_2_ and 105 ± 36.8 µmol H_2_ per g_CN_*x*__ under air, respectively. These results showed that the air-tolerant SR system could be successfully extended to various biomass substrates.

Further expansion to air-tolerant SR of polymer waste using ZnAce : H_2_O_KP_i__ was conducted by utilising ethylene glycol (EG), a monomeric component of polyethylene terephthalate (PET), as a substrate.^31^ H_2_ production coupled with EG oxidation exhibited significant activity of 805 ± 58 µmol H_2_ per g_CN_*x*__ under N_2_ and 769 ± 37 µmol H_2_ per g_CN_*x*__ under air after 18 h in a ZnAce : H_2_O_KP_i__ (6 : 4 v/v) solution (Fig. 2a). Thus, the SR activity of the PET monomer EG was retained under air compared to inert conditions.

In addition, glycerol (Gly), an industrially relevant alcohol and major byproduct of biodiesel, was also used as a substrate for air-tolerant SR (Fig. 2a).^32^ The SR of Gly in ZnAce : H_2_O_KP_i__ showed slightly lower performance under air, but it still achieved a notable value of 513 ± 79.9 µmol H_2_ per g_CN_*x*__, considering that the reaction was performed in the presence of O_2_.

Subsequently, insoluble and robust polymeric substrates such as cellulose, xylan, and PET were subjected to acid hydrolysis pre-treatment using an aqueous H_2_SO_4_ solution at 120–140 °C depending on the substrate (see experimental Section in SI for details) to more completely depolymerise them into soluble monomers, glucose, xylose, and EG (72% conversion yield for PET according to a previous report; Fig. 2b).^33,34^ The pre-treated solutions were then diluted 10 times and directly used for SR. This pre-treatment step ensures suitable reaction kinetics and better accessibility of monomers to the photocatalyst for SR. It significantly improved the SR performances, yielding activities of 445 ± 41.5 µmol H_2_ per g_CN_*x*__ for cellulose, 545 ± 127 µmol H_2_ per g_CN_*x*__ for xylan, and 388 ± 29.1 µmol H_2_ per g_CN_*x*__ for PET, respectively, after 18 h of SR in ZnAce : H_2_O_acid_ (6 : 4 v/v) solution under air.

Intriguingly, the SR performance for cellulose under air was higher than under N_2_, which is likely due to the generation of ROS *via* residual ORR in the reaction mixture, promoting subsequent fragmentation of partially degraded cellulose into smaller, more readily oxidisable intermediates and thereby enhancing the overall SR efficiency. A control experiment was carried out using the radical scavenger 5,5-dimethyl-1-pyrroline *N*-oxide (DMPO) (Fig. S14). The presence of the radical scavenger showed no significant difference between N_2_ and air, supporting the speculation that the higher SR activity for cellulose under aerobic conditions in ZnAce : H_2_O_acid_ (6 : 4 v/v) solution is mainly attributed to ROS-mediated fragmentation of cellulose into oxidisable species, despite the anticipated low ROS levels resulting from inhibited ORR in the aqueous ZnAce solution. Nevertheless, additional mechanistic investigation will be required in future studies to fully understand the increased aerobic SR activity.

**Fig. 3:**
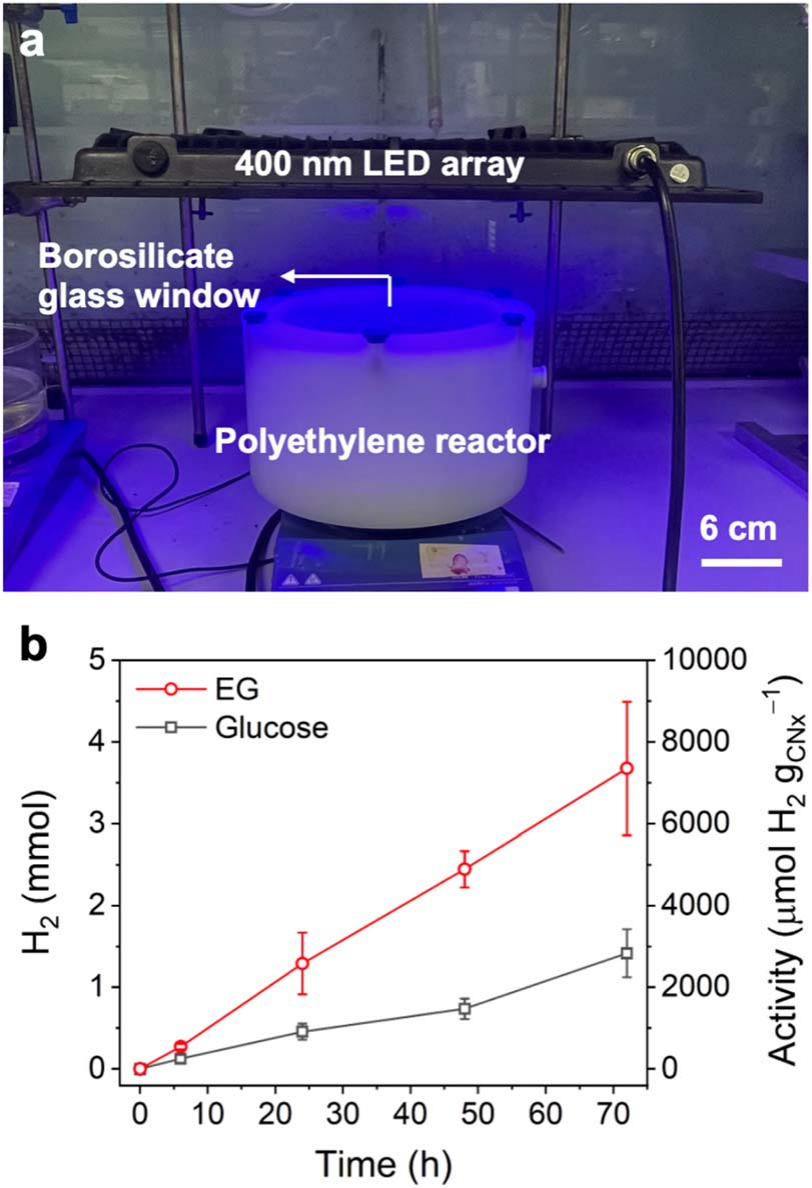
(a) Image of the large-scale setup and (b) H_2_ production using platinised carbon nitride photocatalyst coupled with glucose and ethylene glycol oxidation through upscaled SR under air (*V*_total_ = 300 mL), performed under 400 nm LED irradiation (8 mW cm^−2^); all concentrations were kept constant.

Next, the scalability of the air-tolerant SR system was demonstrated to simulate real-world conditions more closely. The scale-up employed a large polyethylene reactor (0.02 m^2^ irradiation area, 1.7 L reactor volume) with a borosilicate glass window allowing vertical irradiation of a 300 mL SR solution (Fig. 3a).^34^ The same reaction conditions used for the smaller scale aerobic SR were employed, including the concentration of catalyst and substrate as well as the ZnAce/H_2_O_KP_i__ solution composition. Using an LED array as the light source (400 nm, 8 mW cm^−2^), the large-scale air-tolerant SR produced 3.68 mmol ± 0.82 of H_2_ (7.35 ± 1.63 mmol H_2_ per g_CN_*x*__, 36.8 ± 8.15 µmol H_2_ per g_CN_*x*__ cm^−2^) from EG oxidation with an apparent quantum yield (AQY) of 0.52%, and 1.42 mmol ± 0.29 of H_2_ (2.83 ± 0.589 mmol H_2_ per g_CN_*x*__, 14.2 ± 2.95 µmol H_2_ per g_CN_*x*__ cm^−2^) from glucose SR with an AQY of 0.20% under air after 72 h (Fig. 3b), showing that this cost-effective and simple air-tolerant SR system could be successfully applied to large-scale operation.

**Fig. 4:**
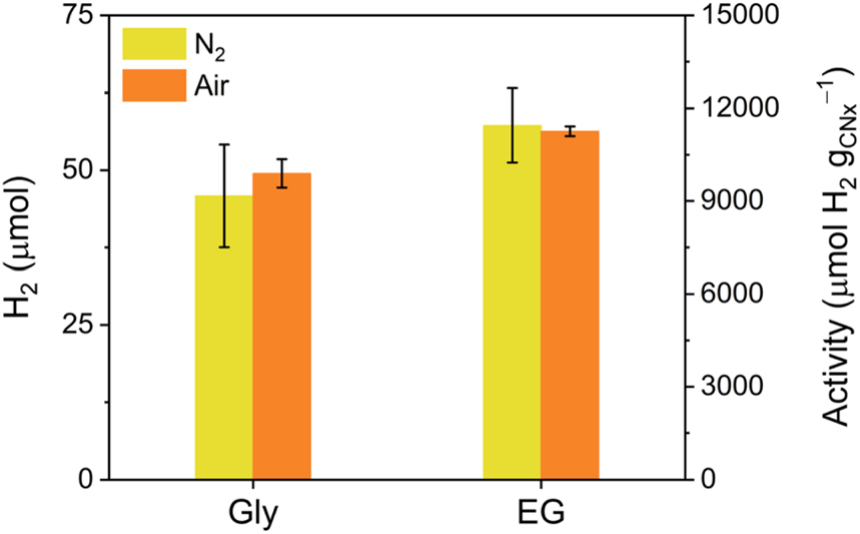
SR performance after 18 h under N_2_ and air using platinised carbon nitride photocatalyst in 3 mL of viscous liquid wastes : H_2_O_KP_i__ (10 mM, pH 4.5) in H_2_O (6 : 4 v/v) solution under simulated solar light irradiation (100 mW cm^−2^, AM 1.5G) at 25 °C.

Given that the high viscosity of the DES reaction medium leads to reduced O_2_ diffusivity and thereby enables the air-tolerant SR, we investigated the feasibility of employing viscous liquid waste directly as a substitute for the DES in order to broaden the applicability of the air-tolerant SR system. In other words, the viscous liquid waste can act directly as both the O_2_-protected reaction medium and the electron donor for SR instead of using ZnAce plus an additional solubilised waste substrate. Accordingly, Gly and EG were subjected to air-tolerant SR using glycerol : H_2_O_KP_i__ (6 : 4 v/v) and EG : H_2_O_KP_i__ (6 : 4 v/v) solutions, respectively (Fig. 4). In these air-tolerant SR systems, exceptional SR performance was achieved, exhibiting 9670 ± 1660 µmol H_2_ per g_CN_*x*__ under N_2_ and 9890 ± 460 µmol H_2_ per g_CN_*x*__ under air for Gly, and 11 400 ± 1200 µmol H_2_ per g_CN_*x*__ under N_2_ and 11 300 ± 158 µmol H_2_ per g_CN_*x*__ under air for EG. Hence, the viscous liquid wastes provided the electron donor for SR in very high concentration and also limited O_2_ diffusivity in the reaction mixture, thereby enabling efficient air-tolerant SR (Table S2). Moreover, Levich plot analysis based on the rotating disk electrode (RDE) voltammetry under O_2_-saturated conditions showed that the introduction of viscous liquids (ZnAce, EG, and Gly) effectively suppressed O_2_ mass transport rate with lowering ORR activity, resulting in sustained photocatalytic SR under aerobic conditions (Fig. S15, S16, Tables S3 and S4).

Beyond monomeric alcoholic waste substrates, polymeric viscous liquid wastes such as polypropylene glycol, polyethylene glycol, and honey were also tested as a reaction medium (Fig. S17). Although the SR activity was relatively low, likely due to the large and complex matrix of the substrate, air-tolerant SR was still achievable with direct use of the polymeric liquid wastes. As a control, methanol, a low-viscosity alcohol, was tested and showed negligible SR performance under air, confirming that high-viscosity liquids are critical to achieve air-tolerant SR.

## Conclusions

We have established an air-tolerant and practical SR system by adjusting the reaction medium to a high viscosity, low O_2_-diffusivity solvent mixed with water. We have identified ZnAce as a robust DES that enables photocatalytic valorisation of pre-treated biomass and plastics, while preventing O_2_ interference with oxidation of organics in solution and simultaneously enabling H_2_ fuel production under air. This SR system maintains its photocatalytic performance under aerobic conditions without significant loss because O_2_ cannot effectively reach the photocatalyst due to the limited mass transport of O_2_ in the viscous solvent mixture. This solvent mixture facilitates a mechanism proceeding *via* direct hole transfer from the photocatalyst to the substrate, resulting in the same oxidation products as those observed in established SR systems operating in aqueous solutions under N_2_. This air-tolerant SR system was applied to the conversion of insoluble polymeric lignocellulosic biomass (*e.g.*, xylan, cellulose) and plastic waste (*e.g.*, PET), where the SR performance can be substantially enhanced by employing acid pre-treatment to break the bulky substrates into soluble organic feedstocks. The scalability of the air-tolerant SR system was validated through scaled-up demonstrations of SR using waste substrates. Notably, the SR system also accommodated viscous liquid waste instead of DES to be used as electron donor and to promote O_2_-tolerance, underscoring its adaptability and versatility. This simple and robust strategy for achieving air-tolerant SR holds potential for practical deployment in biomass and plastic waste treatment with sustainable fuel generation. In the future, low O_2_-diffusivity solvents may also pave the way for air-tolerant organic photocatalysis.

## Author contributions

D. K. and E. R. designed the project. D. K. conducted the main experimental work, including photocatalyst synthesis and characterisation, photocatalysis, product quantification and characterisation, electrochemical analysis, and data analysis. P. K. K. designed the large-scale reactor and conducted acid pre-treatment. D. K. and E. R. co-wrote the manuscript with contributions from P. K. K.

## Conflicts of interest

A patent application covering this work has been filed by Cambridge Enterprise that names all co-authors as inventors (GB2518549.7; 06. Nov. 2025).

## Supplementary Material

SC-017-D6SC01540A-s001

## Data Availability

The data that support the findings of this study are openly available at the Cambridge Data repository: https://doi.org/10.17863/CAM.130162. Supplementary information (SI) is available. See DOI: https://doi.org/10.1039/d6sc01540a.
