# Supplementary material for: Air-tolerant solar reforming of pre-treated biomass and plastics in viscous sustainable solvents
Source: Chem Sci. 2026 May 11;17(27):13416–22. doi: 10.1039/d6sc01540a (PMC13223777; doi:10.1039/d6sc01540a)
Supplement: SC-017-D6SC01540A-s001 [file SC-017-D6SC01540A-s001.pdf]

Supplementary Information (SI) for Chemical Science.
This journal is © The Royal Society of Chemistry 2026

Supplementary information

Air-tolerant solar reforming of pre-treated biomass and plastics in viscous sustainable solvents

Dongseok Kim, Papa K. Kwakye Kwarteng, and Erwin Reisner*

Yusuf Hamied Department of Chemistry, University of Cambridge, Lensfield Road, Cambridge, CB2 1EW, U.K.

Email: *reisner@ch.cam.ac.uk

Experimental methods

Materials

All chemicals were purchased from commercial suppliers and used as received unless otherwise noted.

Synthesis of Cyanamide-functionalised Carbon Nitride ($^{NCN}CN_x$)

$^{H2N}CN_x$ was synthesised by polymerising melamine at 550 °C for 4 h in air, following a previously reported method.[1] Bulk $^{NCN}CN_x$ was obtained by grinding $^{H2N}CN_x$ with KSCN, then subjecting the mixture to calcination under Ar at 400 °C for 1 h, followed by 500 °C for 30 min, using a published procedure.[2] Activated $^{NCN}CN_x$ was prepared by ultra-sonicating bulk $^{NCN}CN_x$ in an aqueous solution for 10 min using a sonication probe (Fisher, Sonic Dismembrator).[3]

Synthesis of Deep Eutectic Solvent (DES)

Four different types of DESs were prepared by using a slightly modified published procedure.[4-6] Reline was prepared by mixing choline chloride and urea in a 1:2 molar ratio, ethaline from choline chloride and ethylene glycol (1:2 molar ratio), glyceline from choline chloride and glycerol (1:2 molar ratio), and ZnAce from zinc chloride and acetamide (1:4 molar ratio). The mixtures were stirred at 80 °C until homogeneous liquid formed.

Physical Characterisation

Samples for X-ray photoelectron spectroscopy (XPS) were dispersed in ethanol and 50 μL then drop-casted (x7) onto clean FTO glass slides, followed by drying. XPS analysis was conducted using a Thermo Fisher Scientific K-alpha+ spectrometer. A micro focused monochromatic Al X-ray source (72 W) was used to examine an area of approximately 400 μm. Data acquisition was performed at pass energies of 150 eV for survey scans and 40 eV for high-resolution scans, with step sizes of 1 eV and 0.1 eV, respectively. Charge neutralisation was ensured by employing both low-energy electrons and argon ions. Due to the surface heterogeneity, three distinct regions were selected on each sample for analysis. Inductively coupled plasma optical emission spectrometry (ICP-OES) measurements were carried out using a Thermo Scientific iCAP 700 spectrometer at the Microanalysis Service, University of Cambridge (Department of Chemistry).

Photocatalytic Experiments

Photocatalytic experiments were carried out in a borosilicate glass photoreactor with a total volume of 7.74 mL, maintained at 25 °C using a water bath. $^{NCN}CN_x$ (5 mg) was first dispersed in 0.5 mL of aqueous solution via ultrasonication. The resulting suspension was then added to

DES/water mixtures containing 10 mM potassium phosphate buffer (pH 4.5), photoreforming substrates (typically 50 mg mL^{-1} glucose unless otherwise specified), ensuring a final volume of 3 mL with a DES:H_2O_{KPi} ratio of 6:4 (v/v). Subsequently, 4 wt% of H_2PtCl_6 was added to the reaction mixture as a proton reduction catalyst with in-situ photo-deposition expected under irradiation. The actual Pt loading after photo-deposition was 0.60 ± 0.03 wt%. The prepared reaction mixture was transferred to the photoreactor, which was equipped with a magnetic stirrer bar and tightly sealed with a rubber septum. For experiments conducted under inert conditions, the reactor was purged with N_2 containing 2% CH_4 as an internal standard for gas chromatography. On the other hand, for photoreforming in air, no gas purging was performed. The reaction system was continuously stirred at 600 rpm and maintained at 25 °C under simulated solar irradiation (Newport Oriel, 100 mW cm^{-2}) with an air mass 1.5 global (AM 1.5G) filter and a water filter to eliminate infrared radiation. The produced H_2 gas was monitored periodically by sampling 50 μL of the headspace and analysing it via gas chromatography.

Large-scale experiment

Home-made large-scale ultra-high molecular weight polyethylene reactor (0.02 m^2, 1.7 L) with a borosilicate glass window was used for the large-scale demonstration.[7] A total of 300 mL of the reaction solution was charged to the large reactor with the same concentration of $^{NCN}CN_x$ (1.67 mg mL^{-1}), H_2PtCl_6 (4 wt%), potassium phosphate buffer (10 mM, pH 4.5), and substrates (33.3 mg mL^{-1}) with a ZnAce:H_2O_{KPi} ratio of 6:4 (v/v) under air. For top-down irradiation, commercially available LED array (400 nm, 8 mW cm^{-2}) was employed, then the produced H_2 gas was monitored periodically for 72 h by sampling 50 μL of the headspace and analysing it via gas chromatography

Quantification and Characterisation of Products

The produced H_2 gas was monitored using a Shimadzu Tracera GC-2010 Plus gas chromatograph (GC) equipped with a barrier discharge ionisation detector, a Hayesep D pre-column, and an RT-Molsieve 5A main column to separate H_2, O_2, N_2, CH_4, and CO. In a typical experiment, 50 μL of headspace gas from the photoreactor was collected using an airtight Hamilton GASTIGHT syringe and injected into the GC for analysis. GC response factors were established by calibration with known amounts of H_2.

Photocatalytic oxidation products were identified using High-Performance Liquid Chromatography (HPLC) following glucose photoreforming for 5 days. The reaction solutions after photoreforming were centrifuged at 8000 rpm for 5 min, after which the supernatant was separated, diluted with an equal volume of water. Then, the solution was injected into a Waters Breeze system equipped with a refractive index detector and a Rezex ROA-Organic Acid H^+ (8%) 300 x 7.8 mm HPLC column, with 5 mM H_2SO_4 as the eluent at a flow rate of 0.5 mL

min^{-1} at 75 °C. In addition, the supernatant was also analysed using proton nuclear magnetic resonance spectroscopy (^{1}H NMR) on a Bruker 500 MHz AVIII HD Smart Probe Spectrometer in D_2O.

Electrochemical analysis

Electrochemical experiments were conducted in a single compartment cell using an Ivium CompactStat potentiostat. A three-electrode configuration with a glassy carbon electrode as a working electrode, a Pt mesh as a counter electrode, and a Ag/AgCl (KCl saturated) as a reference electrode was employed. Linear sweep voltammetry (LSV) was recorded in a DESs (Reline, Ethaline, Glyceline, and ZnAce):H_2O solution (6:4 v/v) containing 10 mM of potassium phosphate buffer (pH 4.5) under N_2. Rotating disk electrode (RDE) voltammetry was performed in a viscous liquids (ethylene glycol, glycerol, and ZnAce):H_2O solution (6:4 v/v) containing 10 mM of potassium phosphate buffer (pH 4.5). The solution was purged with either N_2 or O_2 for 15 mins before the RDE measurement.

Acid pretreatment of polymeric substrates

Acid pretreatment of polymeric substrates was proceeded following a published procedure.[7, 8] For the acid hydrolysis of polymeric substrates, an empty commercial PET water bottle was cut into smaller pieces (1.5 – 3 cm^2) and frozen with liquid nitrogen. A coffee grinder was then used to pulverise the pieces into flakes. 1.5 g of the PET flakes was placed in a round-bottomed flask and 7.5 M H_2SO_4 was added and the mixture heated to 140 °C for 6 h. The resulting mixture was centrifuged and the supernatant collected with 72% conversion yield and ~100 mM of concentration of ethylene glycol. For cellulose and xylan substates, 1.5 g of substrates were added to 11.4 M H_2SO_4 aqueous solution and stirred at 30 °C for 1 h. The yellowish hydrolysates were then diluted to the concentration of 3 M H_2SO_4 and subjected to further heating at 120 °C for 30 mins. The resulting depolymerised solutions were subsequently diluted to the concentration of 0.75 M H_2SO_4 for use in solar reforming experiments.

O_2 sensing

O_2 level in headspace: Amount of O_2 was monitored while photoreforming of glucose in a N_2-filled glovebox using a NeoFox-GT fluorometer and Fospor-R fluorescence oxygen sensor probe (Ocean Optics).

O_2 solubility: O_2 solubility was measured in the dark by immersing a NeoFox-GT fluorometer and Fospor-R fluorescence oxygen sensor probe (Ocean Optics) into the ZnAce/H_2O, glycerol/H_2O, ethylene glycol/H_2O solution mixtures containing 10 mM of potassium phosphate buffer (pH 4.5).

O_2-diffusivity calculation

O_2-diffusivity in the DES:H_2O_{KPi} and viscous liquids:H_2O_{KPi} (6:4 v/v) solutions was evaluated using the Levich equation (eqn. 1):

$$i_L = 0.62nFD^{2/3}v^{-1/6}C^*\omega^{1/2} \quad \text{(eqn. 1)}$$

,where i_L = limiting current density, n = number of electrons for ORR, F = Faraday constant, D = diffusion coefficient of O_2 (diffusivity), v = kinematic viscosity, C^* = bulk O_2 concentration (solubility), and ω = angular rotation speed

The limiting current densities (i_L) for ORR were obtained with a rotating disk electrode (RDE) voltammetry, the kinematic viscosities (v) were measured using an Ubbelohde Viscometer, and O_2 solubilities (C^*) were observed using oxygen sensor probe. Based on these experimentally obtained parameters, diffusion coefficient of O_2 (O_2 diffusivity; D), which is a key factor enabling air-tolerant SR, was calculated as described below:

$$D = \left(\frac{i_L}{0.62nFv^{-1/6}C^*\omega^{1/2}}\right)^{3/2} \quad \text{(eqn. 2)}$$

Apparent quantum yield (AQY) determination

AQY was determined directly using H_2 product results from the large-scale air-tolerant SR experiment with single-wavelength purple LEDs (405 nm) using eqn. 3:

$$AQY\ (\%) = \frac{2 \times Moles\ of\ H_2 \times N_A \times h \times c}{Power\ of\ light\ source\ (P) \times t \times \lambda} \times 100 \quad \text{(eqn. 3)}$$

,where moles of H_2 is the amount of hydrogen measured by GC, N_A is Avogadro's number given as 6.022 x 10^{23} mol^{-1}, h is Plank's constant given as 6.626 x 10^{-34} Js, c is the speed of light given as 3.0 x 10^8 m s^{-1}, power of light source (P) was measured as 8 mW cm^{-2} by using the light meter at the surface of the reaction medium, λ is the wavelength of light, and t is reaction time in seconds. The calculation assumes perfect absorption of photons.

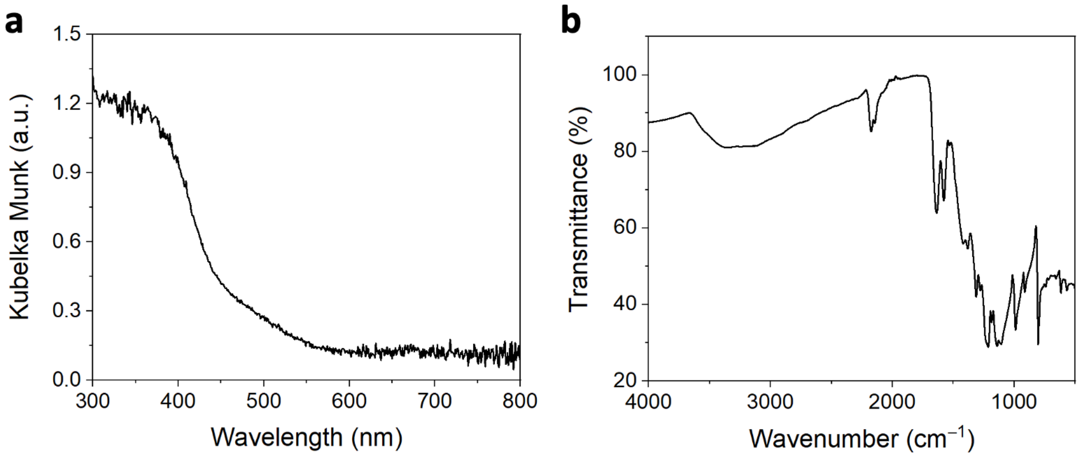

Figure S1. A) UV-Vis diffuse reflectance spectra (DRS) and b) FT-IR spectra of cyanamide-functionalised carbon nitride.

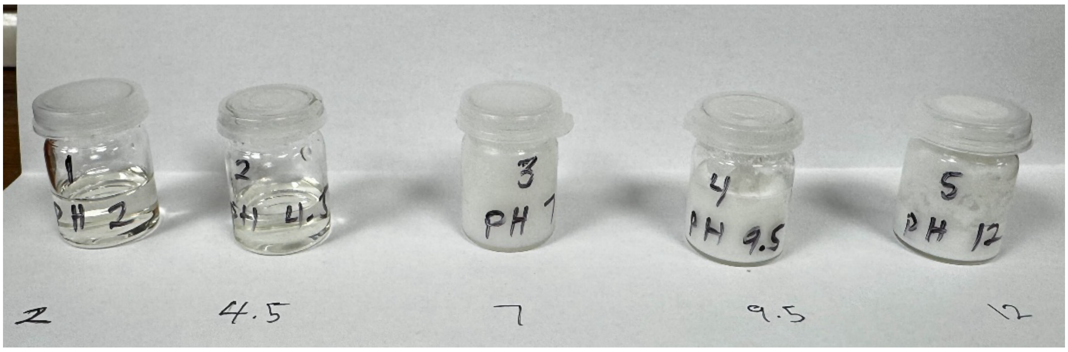

Figure S2. A photograph image of ZnAce/H_2O solutions at different pH values ranging from 2 to 12.

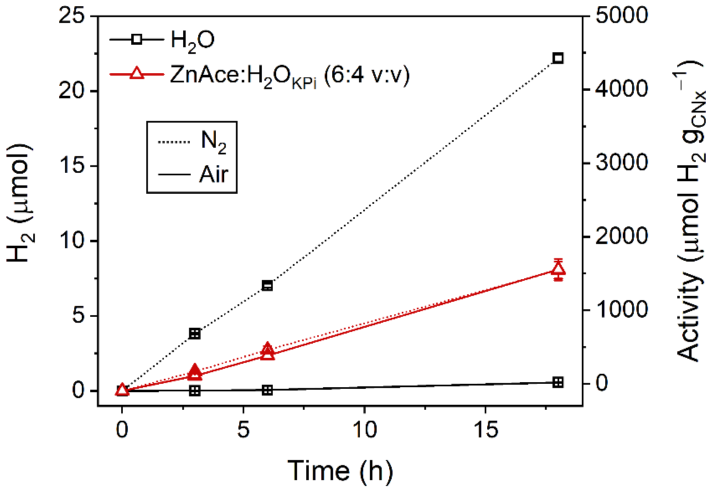

Figure S3. Comparison of the photocatalytic activity of H_2 production in ZnAce:H_2O_{KPi} (6:4 v/v) versus pure KPi aqueous solution (10 mM, pH 4.5) as reaction media. The SR was conducted

under N_2 and air using platinised carbon nitride photocatalyst with glucose (50 mg mL^{-1}) under simulated solar light irradiation (100 mW cm^{-2}, AM 1.5 G) at 25 °C.

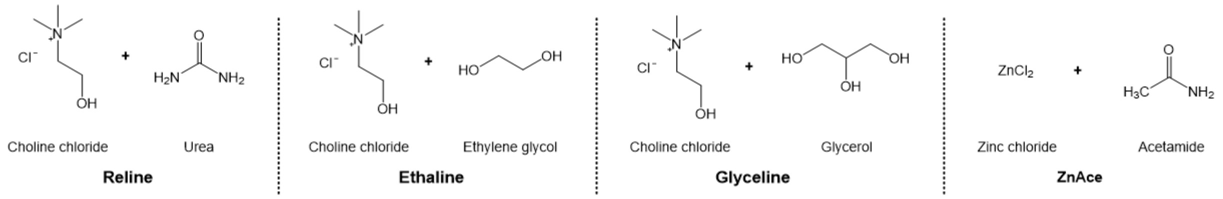

Figure S4. Chemical structures of different types of deep eutectic solvents termed as Reline, Ethaline, Glyceline, and ZnAce.

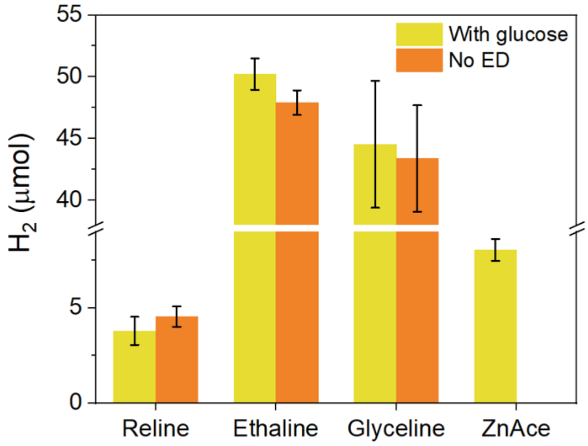

Figure S5. Photocatalytic H_2 production using platinised carbon nitride photocatalyst with different types of DESs in the presence and absence of the electron donor (glucose, 50 mg mL^{-1}) under simulated solar light irradiation (100 mW cm^{-2}, AM 1.5 G) at 25 °C under N_2 in 3 mL of DES:H_2O_{KPi} (6:4 v/v) solution.

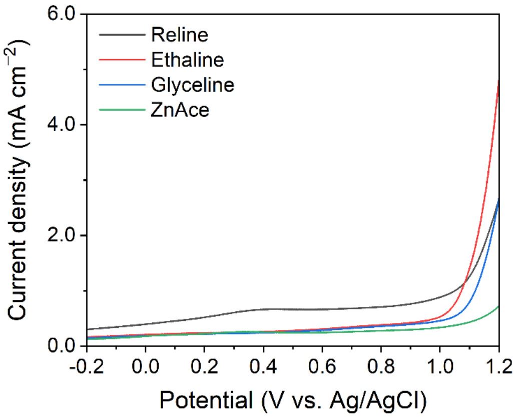

Figure S6. Linear sweep voltammograms of the DES:H_2O solutions (6:4 v/v) under N_2 atmospheres at a scan rate of 50 mV s^{-1} with glassy carbon electrode as a working electrode, Pt mesh as a counter electrode and Ag/AgCl (KCl saturated) as a reference electrode.

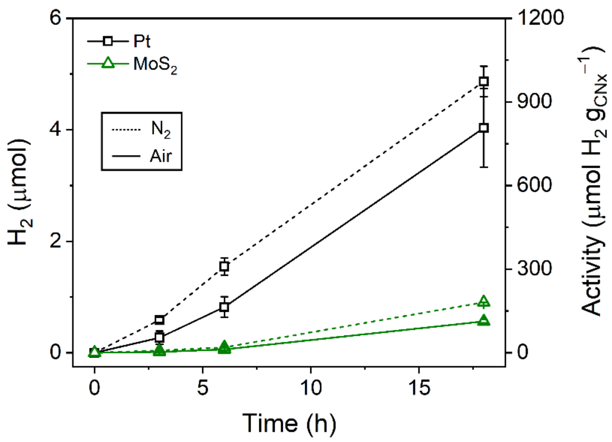

Figure S7. Photocatalytic H_2 production under N_2 and air under simulated solar light irradiation (100 mW cm^{-2}, AM 1.5 G) at 25 °C in ZnAce:H_2O_{KPi} (3 mL, 6:4 v/v) solution containing glucose (50 mg mL^{-1}), platinised carbon nitride photocatalyst (5 mg) and different co-catalysts; Pt (precursor: H_2PtCl_6) and MoS_2 (precursor: $H_8N_2MoS_4$).

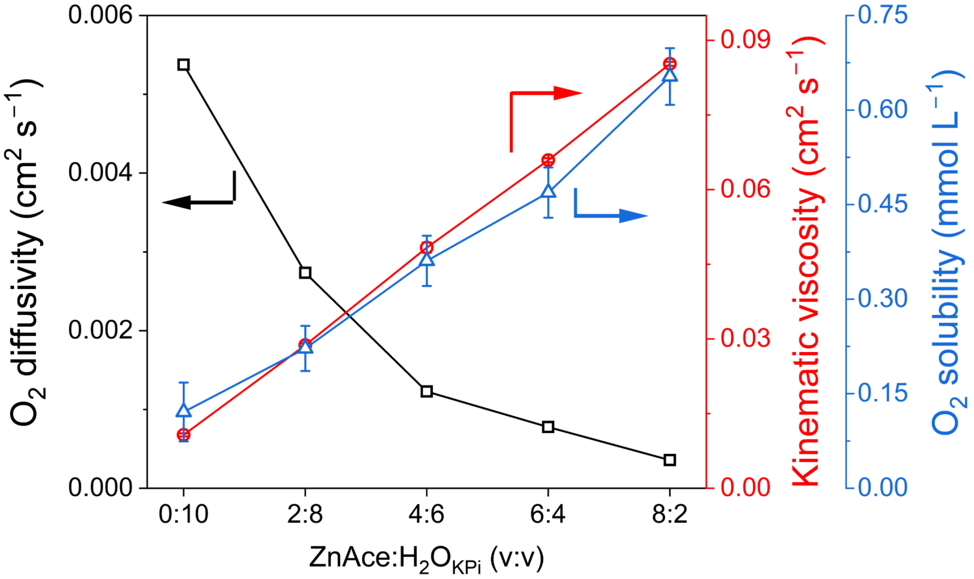

Figure S8. Correlation among kinematic viscosity, O_2 solubility, O_2 diffusivity depending on the ZnAce:H_2O_{KPi} volume ratio.

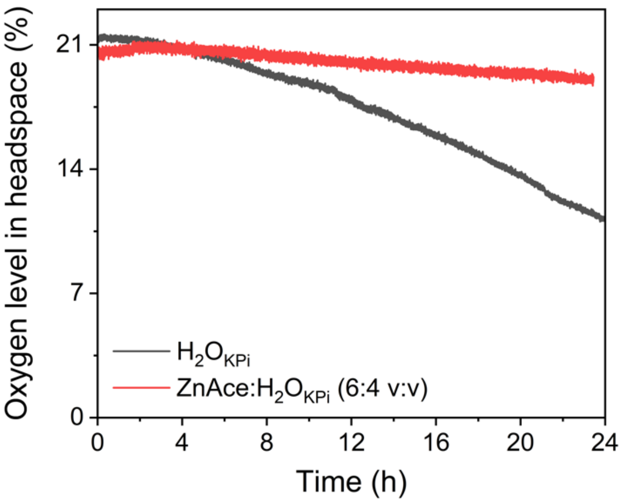

Figure S9. O_2 concentration in the headspace (4.7 mL) during glucose SR under the simulated solar light irradiation (100 mW cm^{-2}, AM 1.5 G) at 25 °C under air, using ZnAce:H_2O_{KPi} (6:4 v/v) or H_2O in the absence of ZnAce.

a

**Reduction**	$2H^+$	+	$2e^-$	→	H_2							
**Oxidation**	$C_6H_{12}O_6$	+	H_2O	→	$C_5H_{10}O_5$	+	HCOOH	+	$2H^+$	+	$2e^-$
**Redox**	$C_6H_{12}O_6$	+	H_2O	→	H_2	+	$C_5H_{10}O_5$	+	HCOOH			

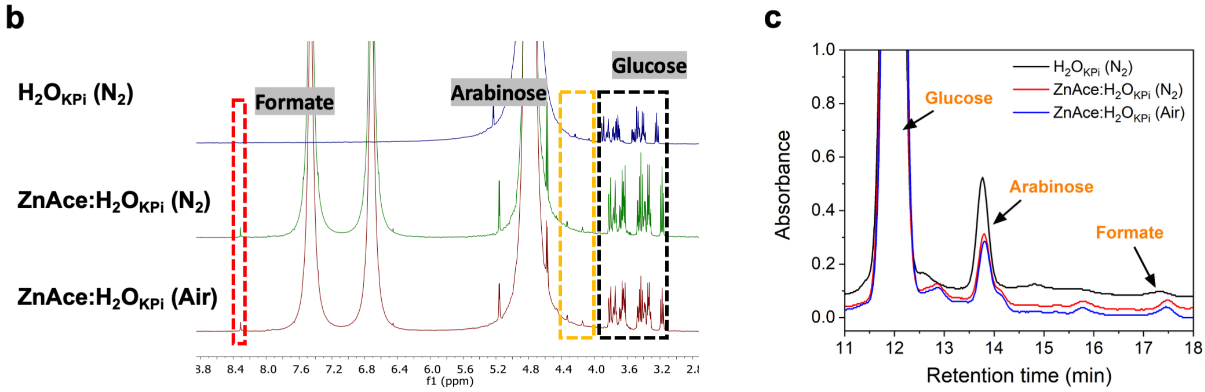

Figure S10. a) Reduction and oxidation half reactions for the redox reaction of H_2, $C_6H_{12}O_6$ (glucose) and H_2O to $C_5H_{10}O_5$ (arabinose) and HCOOH. b) ^{1}H NMR and c) HPLC spectra after solar reforming of glucose. Photoreforming condition: platinised carbon nitride photocatalyst (5 mg), glucose (50 mg mL^{-1}), ZnAce:H_2O_{KPi} (3 mL, 6:4 v/v) solution, simulated solar light (5 days, 100 mW cm^{-2}, AM 1.5 G) at 25 °C.

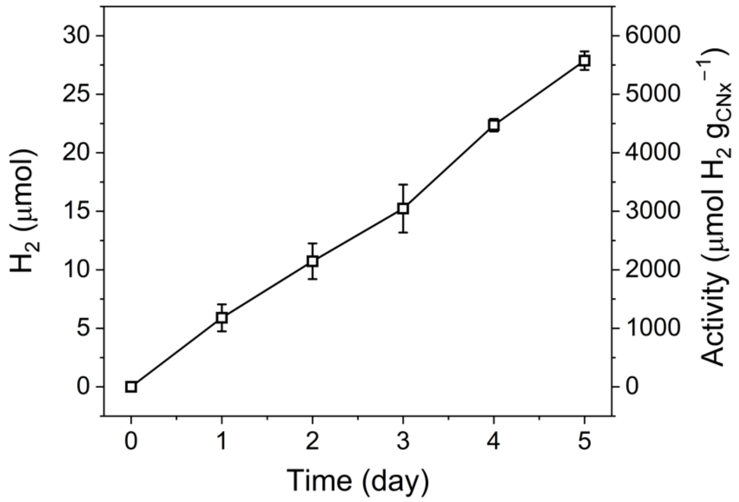

Figure S11. Photocatalytic H_2 production under air using platinised carbon nitride photocatalyst under simulated solar light irradiation (100 mW cm^{-2}, AM 1.5 G) at 25 °C in ZnAce:H_2O_{KPi} (3 mL, 6:4 v/v) solution containing glucose (50 mg mL^{-1}).

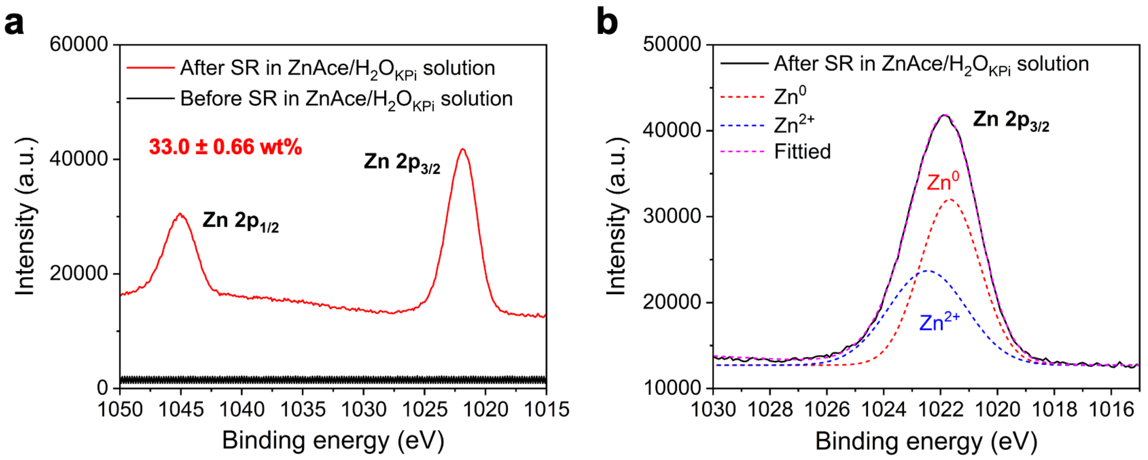

Figure S12. (a) High resolution XPS spectra for Zn 2p before and after SR under simulated solar light irradiation (100 mW cm^{-2}, AM 1.5 G) at 25 °C in 3 mL of ZnAce:H_2O_{KPi} (6:4 v/v) solution under air after 18 h. ICP-OES analysis revealed that the Zn content in the platinised carbon nitride photocatalyst after SR was 33.0 ± 0.66 wt%. (b) Deconvoluted Zn $2p_{3/2}$ spectra after SR showing the presence of both Zn^0 and Zn^{2+} species.

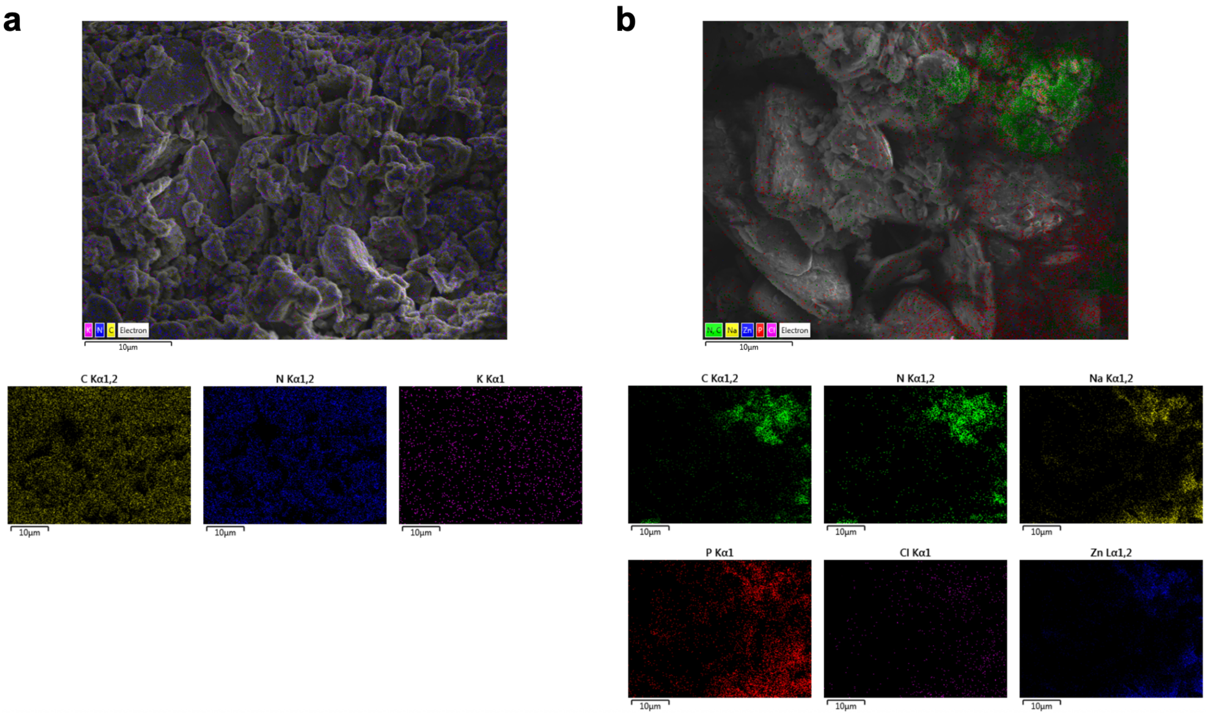

Figure S13. TEM image with energy-dispersive X-ray (EDX) mapping for different elements (C, N, K, P, Na, Cl, and Zn) of platinised carbon nitride photocatalyst after the SR under solar light irradiation (100 mW cm^{-2}, AM 1.5G) (a) in H_2O_{KPi} solution and (b) in ZnAce:H_2O_{KPi} (6:4 v/v) solution for 18 h at 25 °C under air.

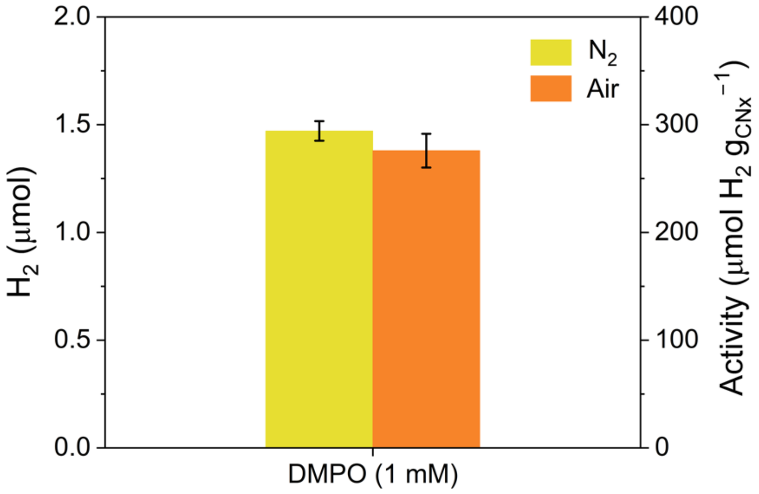

Figure S14. SR performance coupled with H_2 production after 18 h under N_2 and air using platinised carbon nitride photocatalyst and pretreated cellulose in 3 mL of ZnAce:H_2SO_4 (0.75M) (6:4 v/v) solution containing 1 mM of DMPO under simulated solar light irradiation (100 mW cm^{-2}, AM 1.5G) at 25 °C.

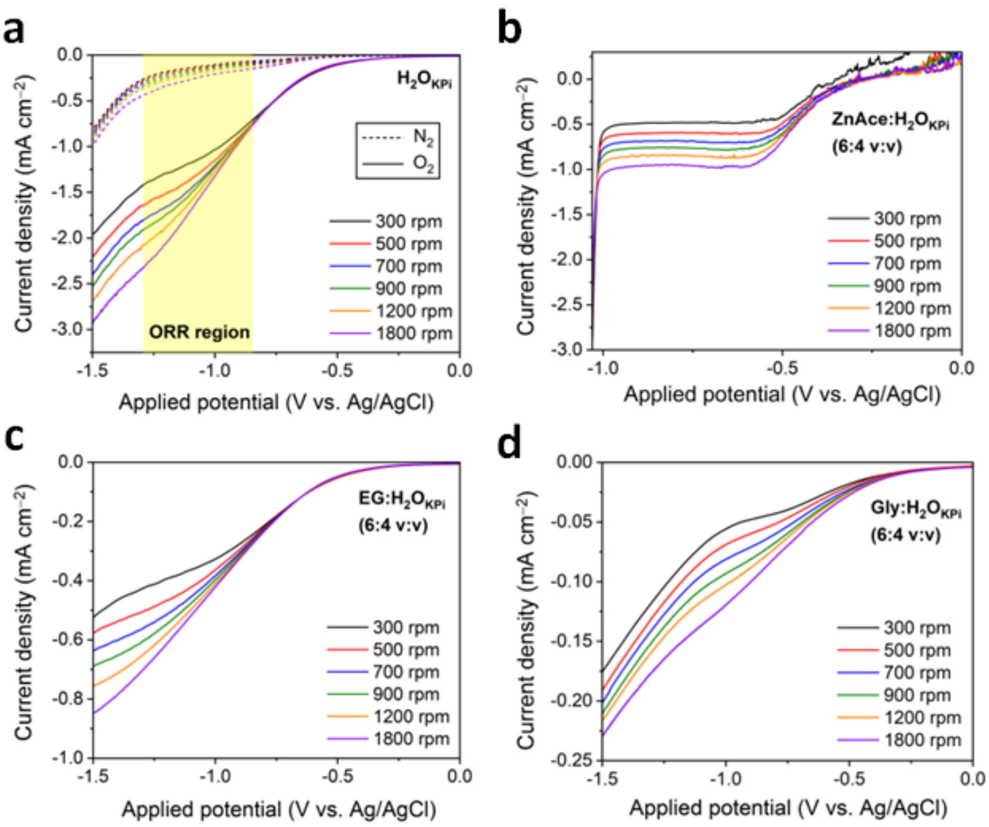

Figure S15. Rotating disk electrode (RDE) voltametric response in the O_2 saturated a) phosphate-buffered (10 mM) aqueous solution, b) ZnAce:H_2O_{KPi} (6:4 v/v) solution, c) EG:H_2O_{KPi} (6:4 v/v) solution, and d) Gly:H_2O_{KPi} (6:4 v/v) solution at a scan rate of 50 mV s^{-1} with Pt mesh as a counter electrode and Ag/AgCl (KCl saturated) as a reference electrode. Short dash plots are conducted under N_2.

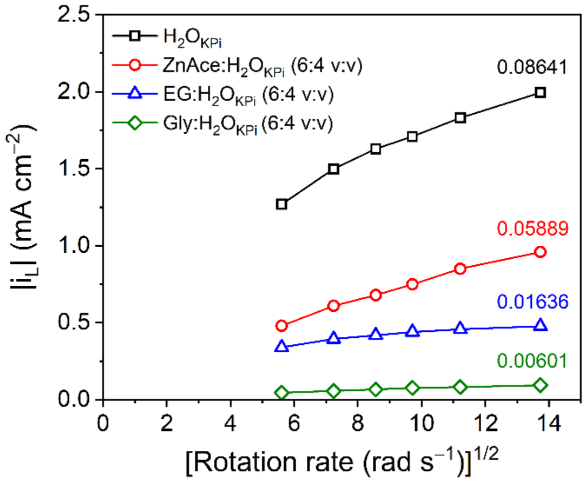

Figure S16. Levich plots of each viscous solvents calculated by using the limited current density of ORR in RDE voltammetry under O_2-saturated condition with corresponding slopes.

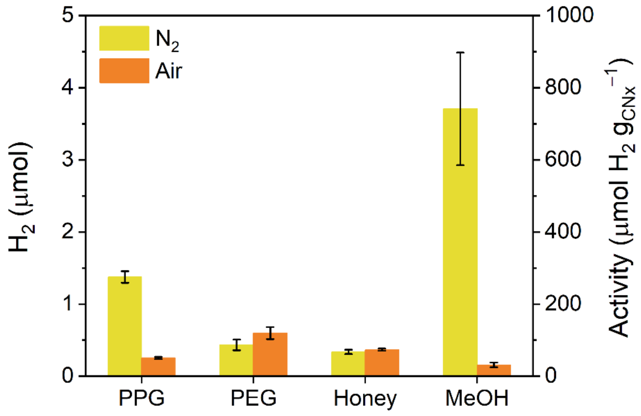

Figure S17. SR performance after 18 h under N_2 and air using platinised carbon nitride photocatalyst in 3 mL of viscous liquid wastes:H_2O_{KPi} (6:4 v/v) solution under simulated solar light irradiation (100 mW cm^{-2}, AM 1.5 G) at 25 °C. *PPG: polypropylene glycol and PEG: polyethylene glycol.

Table S1. Comparison of photocatalytic activity of H_2 production with relevant previous literature.[4, 9-16]

	Entry	Photocatalyst	Electron donor	Deep eutectic solvents	Reaction conditions	H_2 activity under N_2 (μmol H_2 g_{Cat}^{-1} h^{-1})	H_2 activity under Air (μmol H_2 g_{Cat}^{-1} h^{-1})	Quantum yield (%)	Ref.
**Air-tolerant SR**	1	$^{NCN}CN_x$|Pt	800 mM EG<br>280 mM Glucose	ZnAce	Simulated solar light (100 mW cm^{-2}) at RT	89.4 ± 6.33	90.0 ± 7.94	0.52% (EG)<br>0.20% (Glucose)	This work
**Air-tolerant photocatalytic H_2 production**	2	$^{NCN}CN_x$|Pt	400 mM TEOA	Reline	Simulated solar light (100 mW cm^{-2}) at 40 ℃	4410 ± 289	3940.0 ± 258	3.7 ± 1.5	4
	3	TiO_2|hydrogenase	400 mM TEOA	Glyceline	Simulated solar light (100 mW cm^{-2}) at 40 ℃	177 ± 25.1	159 ± 22.6	2.3 ± 0.4	9
	4	EosinY|hydrogenase	150 mM TEOA	-	Simulated solar light (100 mW cm^{-2}, λ>420 nm) at RT	1000 ± 43.4	72.4 ± 14.5	EQE = 1.5 ± 0.08 (under N_2)	10
**Photocatalytic H_2 production using carbon nitride and Pt**	5	EosinY|hCN-G|Pt	10 vol% TEOA	-	Simulated solar light (100 mW cm^{-2}) at RT	16800	-	-	11
	6	SA-Pt/g-C_3N_4-8.7	10 vol% TEOA	-	300 W Xenon lamp at 10 ℃	22700		22.5	12
	7	Oxidized Pt/g-C_3N_4	10 vol% TEOA	-	300 W mercury lamp (43 mW cm^{-2}) at RT	3640		2.4	13
**Solar reforming using carbon nitride and Pt**	8	HGM/CN_x|Pt	400 mM EG	-	1 M KOH , Simulated solar light (100 mW cm^{-2}) at RT	124 ± 16.1	-	-	14
	9	o-g-C_3N_4|Pt	100 mM glucose	-	Simulated solar light (500 mW cm^{-2}) at RT	840 ± 34.0	-	-	15
	10	Pt NPs/PRCN	11 mM glucose	-	300 W Xenon lamp (2.0 W cm^{-2}) at 80 ℃	72.3		-	16

Table S2. Viscosities, O_2 solubility, and O_2 diffusivity in the viscous liquids:H_2O_{KPi} (6:4 v/v).

	Kinematic viscosity (cm^2 s^{-1})	O_2 solubility (mmol L^{-1})	O_2 diffusivity (cm^2 s^{-1})
ZnAce:H_2O_{KPi} (6:4 v:v)	6.59 ± 0.040 x 10^{-2}	0.469 ± 0.040	7.78 x 10^{-4}
EG:H_2O_{KPi} (6:4 v:v)	4.95 ± 0.021 x 10^{-2}	0.488 ± 0.031	7.82 x 10^{-4}
Gly:H_2O_{KPi} (6:4 v:v)	1.17 ± 0.24 x 10^{-1}	0.444 ± 0.030	9.71 x 10^{-5}

Table S3. Limiting current densities (mA cm^{-2}) of ORR in the DES:H_2O_{KPi} solutions.

ZnAce:H_2O_{KPi} (v:v)	300 (rpm)	500	700	900	1200	1800
0:10	1.29	1.49	1.68	1.78	1.97	2.11
2:8	0.68	0.93	1.07	1.23	1.38	1.67
4:6	0.45	0.50	0.65	0.72	0.88	1.12
6:4	0.33	0.42	0.54	0.60	0.73	0.88
8:2	0.27	0.30	0.39	0.43	0.48	0.54

Table S4. Limiting current densities (mA cm^{-2}) of ORR in the viscous liquids:H_2O_{KPi} (6:4 v/v) solutions.

	300 (rpm)	500	700	900	1200	1800
ZnAce:H_2O_{KPi} (6:4 v:v)	0.33	0.42	0.54	0.60	0.73	0.88
EG:H_2O_{KPi} (6:4 v:v)	0.48	0.61	0.68	0.75	0.85	0.96
Gly:H_2O_{KPi} (6:4 v:v)	0.046	0.057	0.067	0.077	0.083	0.094

Supporting References

1. J. Liu, Y. Liu, N. Liu, Y. Han, X. Zhang, H. Huang, Y. Lifshitz, S.-T. Lee, J. Zhong and Z. Kang, *Science*, 2015, **347**, 970-974.
2. H. Kasap, R. Godin, C. Jeay-Bizot, D. S. Achilleos, X. Fang, J. R. Durrant and E. Reisner, *ACS Catal.*, 2018, **8**, 6914-6926.
3. H. Kasap, D. S. Achilleos, A. Huang and E. Reisner, *J. Am. Chem. Soc.*, 2018, **140**, 11604-11607.
4. M. G. Allan, M. J. McKee, F. Marken and M. F. Kuehnel, *Energy Environ. Sci.*, 2021, **14**, 5523-5529.
5. Q. Liu, X. Zhao, D. Yu, H. Yu, Y. Zhang, Z. Xue and T. Mu, *Green Chem.*, 2019, **21**, 5291-5297.
6. S. Hong, X. Sun, H. Lian, J. A. Pojman and J. D. Mota-Morales, *J. Appl. Polym. Sci.*, 2020, **137**, 48385.
7. P. K. Kwarteng, Y. Liu, C. Han, S. A. Bonke, D. M. Vahey, C. Pulignani and E. Reisner, *Joule*, 2026, 102347.
8. T. Yoshioka, T. Sato and A. Okuwaki, *J. Appl. Polym. Sci.*, 1994, **52**, 1353-1355.
9. M. G. Allan, T. Pichon, J. A. McCune, C. Cavazza, A. Le Goff and M. F. Kühnel, *Angew. Chem. Int. Ed.*, 2023, **62**, e202219176.
10. T. Sakai, D. Mersch and E. Reisner, *Angew. Chem. Int. Ed.*, 2013, **52**, 12313-12316.
11. T. T. Dang, K. C. Bhamu, T. Mahvelati-Shamsabadi, T. K. Oanh Nguyen, E. W. Shin, K.-H. Chung, S. H. Hur, W. M. Choi, S. G. Kang and J. S. Chung, *ACS Appl. Nano Mater.*, 2023, **6**, 9825-9838.
12. Z. Zeng, Y. Su, X. Quan, W. Choi, G. Zhang, N. Liu, B. Kim, S. Chen, H. Yu and S. Zhang, *Nano Energy*, 2020, **69**, 104409.
13. D. Q. Dao, T. K. Anh Nguyen, S. G. Kang and E. W. Shin, *ACS Sustain. Chem. Eng.*, 2021, **9**, 14537-14549.
14. S. Linley and E. Reisner, *Adv. Sci.*, 2023, **10**, 2207314.
15. A. Speltini, A. Scalabrini, F. Maraschi, M. Sturini, A. Pisanu, L. Malavasi and A. Profumo, *Int. J. Hydrog. Energy*, 2018, **43**, 14925-14933.
16. H. Zhang, H. Zhao, S. Zhai, R. Zhao, J. Wang, X. Cheng, H. S. Shiran, S. Larter, M. G. Kibria and J. Hu, *Appl. Catal. B. Environ.*, 2022, **316**, 121647.

End of Supporting Information
